# Selections and Abstracts

**Published:** 1916-07

**Authors:** 


### SELECTIONS AND ABSTRACTS

### TO ALL CHAPTERS AND AUXILIARIES.

In connection with the appeal of the Chairman of the Central Committee, Mr. Taft, dated June 26th, 1916, for funds for aid to our soldiers and sailors on or near the Mexican border, which appeal it is to be regretted has been poorly responded to, the following letters showing splendid work done by two chapters in giving needed aid and comfort to soldiers en route to the border, and the special need of financial assistance in carrying on this good work, are published for the information of all concerned.

Press reports indicate that other Red Cross Chapters along the routes followed by the troop trains exerted themselves in like manner early and late to provide comforts and necessities for militiamen.

It is just such good, patriotic and humanitarian work as this that will cause the name of the American Red Cross to be revered by every recipient of the timely aid and comfort given by it, and it is sincerely hoped that these illustrative letters of good Red Cross work may be the means of causing such of our chapters and auxiiaries as have not had an opportunity to take part in such work, to contribute liberally toward its continuance by those chapters which are called upon to do it.

Contributions should be sent to the American Red Cross Washington, D. C., with purposes for which they are intended plainly stated.

ARTHUR MURRAY,
Major-General, U. S. A. (Ret.),
Acting Chairman, Central Committee.

Col. J. R. Kean, Director General,
Department of Military Relief, American Red Cross.
Washington, D. C.

Dear Sir:

Referring to your night lettergram of June 29th, I beg to inform you that this Chapter has established a REFRESHMENT STATION for the benefit of the troops passing through. After a brief twenty-four hours for preparation, we initiated the station by serving 400 men of the first squadron of the California Cavalry with sandwiches, milk, sweet cider, iced tea and watermelons. Since tehn, we have served 2,000 more militiamen with about the same sort of food. Coffee, soup, sausages and such things, are not advisable in this very hot climate. A neighboring Chapter is furnishing us our fruit, also 20 gallons of milk per day, the latter being the most called for and appreciated supply we have.

On inquiring of several detachments what they wanted most, and learning it was a bath, we have arranged with the Y. M. C. A. for the use of their pool and showers, when the length of their stay permits them taking advantage of same.

We, with the assistance of the nearby Chapter, can maintain this station for a short time, but from all I can learn, we will be getting detachments of troops through here all summer. The men have been so very appreciative of this service, simple as it is, and it has served so admirably to break the monotony of their long trip, that if possible, we would like to appeal to some other section of the military relief, to assist us in carrying on this work.

This State, as you doubtless realize, is neither populous or wealthy, but the people are all willing to work their ahrdest, to assist the boys who they feel are primarily protecting them, if they just have material to work with. We very much desire to make this REFRESHMENT STATION a permanent affair, and would appreciate it very much if you would furnish us with further details of organization, and if you will assist us, by advising eastern chapters, who may wish to help their boys on the border, about the work we are doing.

You might be interested in our present arrangements. The Southern Pacific Railway Company and the Wells Fargo Express Company have been most generous with their assistance. The railway company has had a large table arranged in their ice room and have given us the space necessary in this room, for our supplies, which we arrange just before train time, as attractively as possible, on Wells Fargo Express Company trucks. These trucks are well suited for this purpose, as they may be moved along the platform to the most convenient place for serving. On the first truck, we have two large barrels filled with ice water; on the second, sandwiches or cookies, as the case may be, and on the third, iced tea and milk and tobacco. With the watermelons, we find it the best plan to put them in the commissary car of the train for future use.

These arrangement, while very simple, have afforded great delight to all the detachments, and many expressions of gratitude have been offered. Today we received the following note, from the commanding officer of Company "I" First Connecticut Infantry:

"Dear Friends:

"A hearty vote of thanks is extended to you all, from the boys of Company 'I,' for the most courteous and cordial reception extended to them during the short time we were at the station.

"With best wishes for the prosperity of your Chapter.

"Yours sincerely,

H. F. LADBURY, Captain."

Thanking you for any assistance and advise you may give us, and offering our services for any future need which may arise.

Col. J. R. Kean, Director General,
Department of Military Relief, American Red Cross,
Washington, D. C.

My dear Colonel Kean:

Complying with your telegram of June 29th, to our Secretary, calling attention to the necessity of sections and chapters organizing refreshment stations, to contribute to the health and comfort of our troops, beg to say that this Chapter took action at once. We have met practically all of the trains, if not all, passing through here in the day time. We have also met some passing rather late at night and some rather early in the morning. We have furnished ice-cream, sandwiches, soda-water and sometimes cigarettes and tobacco. In other instances, fruit and water-melons have been furnished. Our people have responded generously to this cause. The Chamber of Commerce contributed $250. We appoint a chairman to meet all the trains passing through each day. These ladies collect money to purchase the supplies furnished. I think all the troops have been very much pleased at the attentions thtus shown them. I am sending you some clippips from our local papers in regard to the matter. We have a lady chaiman to meet every train coming through up to and including next Monday. If any more troops come after that we will have another chairman and hope to meet all of them. All of the troops have expressed great appreciation, and I feel sure that they will kindly remember this city and its people.

---

### AMERICAN RED CROSS DIRECTIONS FOR SHIPPING SUPPLIES TO TROOPS ON MEXICAN BORDER.

In connection with the mobilization of our troops on our Southwestern border, the American Red Cross has undertaken to collect and distribute soldiers' gifts and other relief supplies, and the various express companies have cordially co-operated in this humanitarian work and will accept such shipments at TWO-THIRDS of the regular rate.

### SUPPLIES NEEDED.

Supplies contributed should fal lwithin the following list only:

Reading matter, games, comfort bags, pajamas, cotton socks (medium weight, large sizes), towels, pipes and smoking tobacco, cigarettes, electric fans (to hospitals), chewing gum, chocolate in tin boxes, hard candies, instantaneous coffee, evaporated cream and canned fruits and other delicacies in tins.

Surgical dressings and hospital supplies will be acceptable if made according to directions in Red Cross Circular No. 115. (For copies of same address "Bureau of Supplies, American Red Cross," Washington, D. C.)

### SUPPLY DISTRICTS.

Depots for the collection and distribution of supplies have been established at various points, as follows:

1. Red Cross Supply Depot, Bush Terminal No. 19, South Brooklyn, N. Y.

2. Red Cross Supply Depot, 219 East Second Street, Cincinnati, Ohio.

3. Red Cross Supply Depot, Clearing, Argo District, Chicago, Ill.

4. Red Cross Supply Depot, care Montgomery, Ward & Co., Kansas City, Mo.

5. Red Cross Supply Depot, Denver, Col.

6. Red Cross Supply Depot, care Mr. A. B. C. Dohrmann, San Francisco, Cal.

7. Red Cross Supply Depot, care Mayor W. H. Adamson, Douglas, Ariz.

8. Red Cross Supply Depot, 516 San Francisco Street, El Paso, Texas.

9. Red Cross Supply Depot, Avenue E and Fourth Street, San Antonio, Texas.

Contributions should be PREPAID to the appropriate Depot, wence shipments will be forwarded, without further charge to the contributor, to the troops at the font.

Enquire of local express agent as to Depot to which YOUR supplies should be sent.

N. B.—Supplies cannot be accepted which are intended to be forwarded separately to designated individuals. Those who wish to send packages to particular persons are advised to employ the parcel post or express service, such service being at REGULAR rates. Supplies inttended for designated companies or regiments may be accepted for forwarded with the understanding that if such delivery is found to be impracticable these supplies may be donated to other troops.

ARTHUR MURRAY,
Major General, U. S. Army,
Acting Chairman, Central Committee,
American Red Cross.

---

With the experience of a surgeon who is fresh from active service with the medical corps of the German army behind it, a base unit at the German Hospital in New York is being organized under the American Red Cross. Its director is Dr. Frederick Kammrer.

Dr. Kammerer has just withdrawn from active service with the German army and brings to his new task the very valuable knowledge gained at the Germon front in the European war zone.

National Headquarters of the American Red Cross in Washington have just received the following cablegram from the Central Committee of the Russian Red Cross in Petrograd:

"On July 8 about 9:00 o'clock in the morning the hospital ship Vperiod of the Russian Red Cross having all the external marks required by the Hague Convention was torpedoed and sunk about thirty-two leagues from Batum near the village of Vitse (on the Black Sea) by an enemy submarine. There were eight deaths and seven wounded among the crew and sanitary personnel of the ship. All hostile governments had been notified of the equipment of the Vperiod as a hospital ship. All possibility of a mistake is excluded. We protest with most profound indignation against this new crime."

---

### ACID AUTOINTOXICATION IN INFANCY AND CHILDHOOD.

By John Lovett Morse, A.M., M.D., Boston.

Professor of Pediatrics, Harvard Medical School; Visiting Physician, Children's Hospital; and Consulting Physician, Infants' Hospital and the Floating Hospital, Boston.

Beta-oxybutyric acid, diacetic acid and acetone may be grouped together under the collective name of the acetone bodies." Their relation in the order of formation is probably beta-oxybutyric acid, diacetic acid and acetone. The acetone bodies are due to one common cause, that is, a peculiar inhibition of the oxidative processes. The failure of oxidation is not due to lack of oxygen, but to some local interference with oxidation. The excretion of beta-oxybutyric acid represents a much more advanced degree of oxidative perversion than the excretion of acetone or diacetic acid.

Origin of the Acetone Bodies. Beta-oxybutyric acid can be formed only from fats, by degradation, or by synthesis from bodies containing two or three carbon atoms. In the latter case it would be immaterial whether these bodies were a product of the disassimilation of albumen or of fat. It is a one-sided view to consider only the fats or only the proteins in this connection. The phenomenon of acetonuria with its many modifications is easily comprehensible if it is understood that acetone is a synthetic product derived from certain bodies which contain few carbon atoms and which may be derived from different sources. Normally the fragments of the proteins and fat molecules which contain but a few carbon atoms undergo further oxidation. This occurs, however, only in the presence of a sufficient quantity of carbohydrates. The explanation probably lies in the fact that the carbohydrates contain so much oxygen. A portion of this oxygen is presumably liberated when the carbohydrates are broken down and is used for the oxidation processes.

Acetone is normally present in the urine in small amounts. Diacetic acid and beta-oxybutyric acid are never present under normal conditions. The excretion of acetone is increased both in health and disease if the diet consists exclusively of meat and fat. This increase is not due simply to starvation, because the acetonuria persists even if the caloric value of the food is brought up to normal by the addition of fat. The addition of a small amount of carbohydrates under these conditions does not prevent the excretion of acetone in the urine. If, however, sufficient carbohydrates are added, the acetonuria ceases. The addition of fat to an ordinary diet does not cause acetonuria. The absence of carbohydrates is also necessary.

To sum up, the acetone bodies are formed from fat in the absence of carbohydrates or when the metabolism of the carbohydrates is abnormal. They may also, under certain conditions, be formed from protein, the amount formed from protein, being, however, relatively unimportant in comparison with that formed from fat.

The acetone bodies are not formed in the digestive tract. This is proved by the fact that the administration of the acetone modies by the mouth causes no symptoms.

Action of the Acetone Bodies. The deleterious effect of the acetone bodies is not due to any specific toxic properties which they possess, but to their acid character in general and to the power which they possess as acids in withdrawing alkali from the organism. It must be clearly understood that the acetone bodies are in themselves not essentially toxic and that they can prove harmful to the body only by removing the bases. Under normal conditions there is a preponderance of bases over acids in the body. When acids are formed in, or introduced into, the body they have a deleterious influence upon it. The body has, however, a very efficient mechanism to guard against this deleterious action. The reaction of the body is regulated through the blood. Acids displace bicarbonate of soda from the blood and set carbon dioxide free. The excess of carbon dioxide is removed through the lungs. A neutral salt is left, which is removed by the kidneys. They excrete acid phosphate and save the base. The proteins, being amphoteric, are also able to neutralize a portion of the acid. Ammonia is also formed from the fixed proteins of the body and aids in the neutralization of acids.

Etiology of Acid Formation.—In acid intoxication an accumulation of acids occurs as the result of the failure of elimination to keep pace with the production of acids. It may be due to an overwhelmingly rapid production of acids when the eliminating mechanism is normal, as in diabetes, or to the failure of the eliminating mechanism to dispose of a normal amount of acid, as in some case sof nephritis. In the latter instance the urine will contain little or no excess of the acetone bodies. When large amounts of an alkali are given when there is an acid intoxication from the overproduction of the acetone bodies, the elimination of these bodies in the urine may be increased from the setting free of the acetone bodies and the urine contain more of the acetone bodies, although the amount of the acetone bodies in the system is diminished. It is evident ,therefore, that the amount of the acetone bodies in the urine does not necessarily show whether or not these bodies are accumualting in the organism.

Lactic acid, which can be formed from both carbohydrates and protein, may probably also be a cause of acid autointoxication. Evidence in favor of this assumption is that lactic acid is a constant constituent of the blood after exercise, and in dogs appears in the blood after the cramps produced by strychnine or phosphorous poisoning.

ACETONE BODIES IN THE URINE. It has already been stated that under normal conditions the urine contains a minimum amount of acetone. This amount is so small, however, that it is not recognizable by the ordinary reagents. The acetone bodies appear in the urine within a short time after the cessation of the taking of food. The residual carbohydrates in the adult are used up after the fifth or sixth day, after which time the carbohydrates must be derived from the breaking down of proteins. The amount of carbohydrates which ca nbe formed from the metabolism of protein is relatively small and is not sufficient to prevent the abnormal metabolism of fat. The reserve of carbohydrates in infancy and early childhood is relatively smaller than in the adult. Therefore, the acetone bodies should appear more quickly in starvation in early than in adult life. I am not familiar with any data which show how soon after the withdrawal of food the acetone bodies may occur in the urine at this age, but from clinical experience I am inclined to believe that they rarely occur from this cause under forty-eight hours. It is probable that when there is an abnormal accumulation of fat in the parenchymatous organs, as is often the case in disturbances of nutrition in infancy, that the formation of the acetone bodies may occur earlier and be greater in amount.

Considerable quantities of acetone and diacetic acid are regularly found in the urine in a large number of diseases accompanied by high fever, both during the course of the disease and during convalescence. These bodies are especially common in the urine in pneumonia. Holt, for example, found that 70% of babies ill with lobar pneumonia showed acetonuria. The acetone bodies are also very frequently found in diseases of the gastrointestinal tract, especially when there is a diminished ingestion of food and a disturbance of the digestion of the carbohydrates.

Few who have not examined the urine of children regularly for the presence of acetone and diacetic acid realize how frequently they are present. It is a outine procedure at the Children's Hospital, in Boston, to examine the urine for acetone and diacetic acid, as well as for albumin and sugar. They are found so often that no attention is paid to their presence, unless there are some symptoms of acid intoxication. Holt recently stated that acetone was found in 30% of the urines of 200 consecutive young children at the Babies' Hospital, in New York. A pediatrician in Boston told me the other day that acetone was found in 70% of all the urines which were brought to his office during the past year. It is evident, threfore, that the mere presence of the acetone bodies in the urine in children, while it denotes an abnormal condition of the metabolism, does not justify the assumption that the child is suffering from acid intoxication in the absence of other symptoms of this condition.

Diagnosis of Acid Intoxication. How, then, is acidosis, or acid intoxication, to be recognized? It must, of course, under ordinary clinical conditions be recognized by finding the acetone bodies in the urine. It must be remembered, however, that the mere presence of a small, or even of a considerable, amount of these bodies does not justify a positive diagnosis of acid intoxication. Better tests are the examination of the blood for the presence of abnormal acids, the determination of the amount of ammonia excreted in the urine and its relation to the total nitrogen excretion, and the determination of the carbon dioxide coefficient in the expired air.

The most characteristic symptom of acid intoxication in infancy and childhood, outside of the presence of considerable amounts of the acetone bodies in the urine, is a peculiar type of dyspnea or, rather, hyperpnea, without cyanosis. The peculiarities of this type of respiration are that the rate of the respiration is increased, and that both inspiration and expiration are prolonged, the normal relation between the two being preserved. Another evidence of acid intoxication which is very frequent in infancy is a diminution in the output of urine, complete anuria being not at all uncommon. It is possible that in such instances the primary cause of the accumulation of acids is the disturbance of elimination through the kidneys. Vomiting is very common in acid intoxication in both infancy and childhood. There is, however, nothing characteristic about the vomiting in acid intoxication, and vomiting occurs in many other conditions. Vomiting, therefore, even when excessive, does not of itself justify a diagnosis of acid intoxication. Acid intoxication is not infrequently associated with diarrhea. There is, however, nothing characteristic about the diarrhea. In many instances of acid intoxication, moreover, the children are constipated. The breath not infrequently has a peculiar aromatic odor. This odor is, however, not always present and is decidedly difficult of recognition. The cheeks are often flushed and the lips of a peculiar cherry-red color. There is often, also, a white line about the mouth. None of these signs are, however, pathog-acid intoxication is a much less simple matter than would appear from the statements of many physicians and of the newspapers.

Acid Intoxication in Recurrent Vomiting. It has been recognized for some years that certain cases of what is commonly known as recurrent, periodic, or cyclic vomiting are associated with the presence of the acetone bodies in the urine. In most of these instances it is probable that the acetone bodies are simply a manifestation of the inanition resulting from the vomiting and the consequent starvation. In others it is presumable that the symptoms are due to an acid intoxication. The reason for this presumption is that in certain instances the acetone bodies are found in large amounts in the urine at the very beginning of the symptoms, at a time when they could not possibly be due to inanition. Moreover, the symptoms cease with the disappearance of the acetone bodies.

Acid Intoxication in Diarrhea. The acetone bodies are also found not infrequently in the course of severe diarrheal conditions in infancy and early childhood. The majority of infants with severe diarrhea die, however, without any evidence of acid intoxication, either in the urine or symptomatically. On the other hand, a certain number of infants with severe diarrheas not only show large amounts of the acetone bodies in the urine, but show the typical hyperpnea and have a marked diminution in the output of urine. The majority of these babies die. In many of these instances, however, the symptoms of acid intoxication may be relieved by suitable treatment, but the babies die, after a lapse of twelve or twenty-four hours, without further symptoms of acid intoxication. It seems evident that in these cases the acid intoxication is a secondary manifestation and not the cause of the disease, although it may be in certain instances the cause of death.

Acid Intoxication in Infections. The acetone bodies are also found in large amounts in association with vomiting, with or without diarrhea, in infants and young children in the course of, or following, various infectious diseases. These diseases may of themselves not be serious. They are very likely to be relatively mild infections of the nasopharynx, tonsillitis, or "grippe." In these instances the symptoms of vomiting and prostration are entirely out of proportion to the severity of the original infection. When death occurs in these cases, it may be that it is due to acid intoxication. If so, it is evident, however, that the original cause of the disease is not acid intoxication and that the acid intoxication is due to some disturbance of the metabolism resulting from a toxemia or from a bacterial infection.

Epidemics of Acidosis. It is because of the occurrence within a short time of a number of cases of acid intoxication of this type, secondary to the infectious diseases, that we have heard so much in the past few years of epidemics of acidosis. It is evident, however, from what we know of the etiology of acidosis, that an epidemic of primary acidosis is an impossibility. When a series of cases of acid intoxication occurs, the explanation is simply that a number of cases of secondary acid intoxication have happened to occur in the course of an epidemic of some other disease. Much attention was attracted, two years ago, by a so-called epidemic of acid intoxication in Concord, N. H. This series of cases has been described by Metcalf. In

70% there was a well-defined infection of the respiratory tract or its adjacent cavities. In the others there was no suggestive etiologic point. A great deal has appeared in the newspapers during the past few weeks about an epidemic of acidosis in Boston and vicinity. I have seen a few of these cases in consultation and have known about a good many more. In the vast majority of these cases no examination of the urine was made. To the best of my knowledge and belief, no examination of the blood, of the ammonia content of the urine or of the carbon dioxide coefficient of the expired air was made in any case. In the vast majority of the cases, therefore, there was no scientific justification whatever for the diagnosis of acidosis. It is probable also that in the vast majority of instances the diagnosis of acidosis had no reasonable clinical justification. In fact, it is probable that very few of the cases reported as acidosis had acidosis at all. How little basis there could have been for the diagnosis in many instances is evident when we consider, for example, that many of the so-called cases of acidosis occurred in the practice of a physician who, according to the newspapers, saw six hundred cases in his office and made nine hundred house visits in one week. If we stop to reckon how much time he could have given to each visit, it is evident that his examinations must have been very superficial and that the work in his laboratory must of necessity have been very limited. Further evidence of the general carelessness in diagnosis at this time is the fact that three cases were admitted to the Boston Children's Hospital on the same day with the diagnosis of acidosis. One of these children had pneumonia and another appendicitis. The third did have acetone in the urine, but was sitting up and crying for eggs the next morning.

It is true, nevertheless, that some very strange and unusual cases occurred during this period. It is also true that in a number of these cases large amounts of the acetone bodies were found in the urine and that, when these cases recovered, the acetone bodies first disappeared from the urine, while in the fatal cases they persisted. The histories of a few of these cases, which I myself saw are as follows:

Case 1. A girl, of twenty months, vomited and had loose stools, Dec. 23. She was given bicarbonate of soda and did not vomit for the next two days, but continued to have four or five loose, greenish stools daily. During this time she took only a little barley water. She began to vomit again on the morning of December 26, and continued to vomit constantly from that time on. The movements from the bowels became very frequent and loose. It was impossible to tell how many she was having, however, because she was having treatment by seepage. She was seen at noon, Dec. 27.

She was conscious, but very feeble. She had evidently lost considerable weight. She was a little dusky. The heart was feeble and there was evidences of edema of the lungs. The abdomen was full, but otherwise negative. The liver and spleen were not palpable. There was nothing else abnormal. The urine had not been examined for the acetone bodies. The bicarbonate of soda which she was taking by mouth and rectum was continued and glucose added to it. She died during the afternoon. There was no autopsy.

Case 2. A boy, of 28 months, that had been perfectly well and had eaten nothing unusual, suddenly began to vomit at 5 p. m., Dec. 31. He continued to vomit about every fifteen minutes up to the time that he was seen by his physician, two hours later. He was then profoundly collapsed, pale and with cold extremities. The temperature in the groin was 96.4 degrees F. He was given bicarbonate of soda by mouth, but this was vomited. When I saw him at 10:30 p. m. he had rallied a little. His color was good. There was no odor of acetone in the breath. He was conscious, but much prostrated. The physical examination was absolutely negative. He vomited a little watery mucus during the examination. The urine, which was obtained a few hours later, contained large amounts of the acetone bodies. Bicarbonate of soda and glucose were given by the mouth, but were not retained. He continued to vomit up to three o'clock the next morning. Castor oil and calomel, which had been given during the night, brought away several very large, foul movements about this time. The vomiting immediately ceased and he was apparently rapidly getting well. Twenty-four hours later, however, all the symptoms returned, and he died in a few hours. The autopsy showed a marked general acetone odor of the body. The tonsils were enlarged and contained numerous small foci of pus. The liver was fatty and the kidneys somewhat degenerated. There was marked edema and injection of the brain. Streptococci were found in the tonsils, brain and spinal fluid. They were not found in the other organs or in the blood. Staphylococci were also found.

Case 3. A boy, nineteen months old, was a little out of sorts on Dec. 27. He seemed all right, however, on the 28th. He had a good stool the morning of the 29th as the result of castoria given the night before. He did not seem very well during the morning, and his physician was called at 2:30 in the afternoon. He prescribed bicarbonate of soda, which was given at once. He vomited about 3 p. m., but not again until 7:30 p. m., when he began to vomit every few minutes and to have loose, watery, foul stools. He was seen at 9:45 p. m. The physical examination was entirely negative. There was no aromatic odor to the breath. He vomited a large amount of watery material several times and had two large, watery stools, slightly brownish and with a very foul odor, during the examination. He continued to vomit and have watery movements throughout the night, and at 6 a. m. the next morning had a convulsion and died. The urine was not examined.

In this instance the symptoms were typical of cholera infantum, and at any other time no other diagnosis would have been considered.

Case 4. A boy of thirteen months, perfectly well, except that he had had a slight cold for several days, began to vomit at 4:50 p. m., Dec. 30. He vomited three times. He was given castor oil at once and thoroughly cleaned out. He was given bicarbonate of soda during the night and repeated enemata of bicarbonate of soda. He was started at once on barley water, to which lactose was soon added. The next day he had many stools, which were not foul. His temperature was

104 degrees F. in the morning, 106 degrees F. in the afternoon and 103 degrees F. at midnight. There was no recurrence of the vomiting. The treatment was continued. He was much better on the morning of Jan. 1, and rapidly recovered. His physical examination was always negative. His breath did not smell of acetone. The urine was not examined. There is no proof, therefore, that this child had acid intoxication. Acid intoxication would probably not have been thought of at any other time. Nevertheless, he responded to the treatment which has seemed most effectual in this condition, that is, the immediate emptying of the intestinal tract and the administration of alkalis and carbohydrates.

Case 5. A girl, sixteen months old, was a little restless on January 21. She began to vomit on the 22nd and had two loose stools. Her physician was called on the 23rd. He found a considerable amount of acetone in the urine, ordered bicarbonate of soda and carbohydrates by the mouth and gave enemata of bicarbonate of soda. She vomited several times during that day, her temperature gradually going up to 102.4 degrees F. There was acetone in the urine the morning of the 24th. She was seen at 4 p. m. on the 24th. She had evidently lost much weights, her cheeks were flushed and her lips cherry red. Her respiration was rapid and deep. The physical examination was otherwise negative. She was given castor oil and put on barley water with dextri-maltose, while the bicarbonate of soda was continued. She promptly recovered.

Pathology. Little or nothing is known as to the pathological changes in this condition. Degenerative changes have been found in the parenchymatous organs, but nothing in any way pathognominic. In two cases in which this diagnosis had been made, which were examined recently in Boston by Dr. Magrath, the bodies both had a marked acetone odor, the tonsils showed definite evidences of infection in both, the livers were fatty and the kidneys somewhat degenerated. There was marked edema of the brain in both instances. Streptococci were found in the tonsils, brains and cerebrospinal fluid in both. In one the staphylococcus aureus was also present. No bacteria were found in the other organs or in the blood in one case, and no cultures were made from the other organs in the other case.

TREATMENT. The cause of the symptoms in acid intoxication being the withdrawal of bases from the organism as the result of an excess of acids in the system, it is evident that the treatment indicated is the introduction of alkalis into the system to neutralize the acids in the body and in this way to prevent the further withdrawal of bases from the body and to allow the reaccumulation of the bases which have been abstracted from the body. The best alkali to use is the bicarbonate of soda. This may be given by the mouth, by the rectum, subcutaneously or intravenously. It is preferable to give it by the mouth if it can be retained. It is best given in water, but, if desired, the taste may be disguised by orange juice or grape juice. It is seldom wise to make the concentration of the solution stronger than 1:20. It is usually better to make it 1:30 or 1:60. Stronger solutions are almost certain of themselves to cause vomiting. The general feeling is that as much soda should be given as possible. It is very probable, however, that excessive amounts of soda may of themselves cause vomiting and diarrhea, and perhaps poisoning. I am very confident that in a considerable number of instances in which I have been called in consultation to see cases of supposed "acidosis," the vomiting and diarrhea were the result of excessive doses of bicarbonate of soda and not symptoms of acid intoxication. In one instance of which I know, in which a baby of fourteen months received three ounces of dry bicarbonate of soda in twelve hours, the baby was collapsed and almost moribund, apparently as the result of the large amount of soda. Other alkalis may be used, but are, as a rule, not as satisfactory as bicarbonate of soda.

Bicarbonate of soda should also be given by the rectum. The solution may be stronger when it is given in this way. The solution is more often retained when it is given by seepage than by enema. Seepage is, in my experience, the more satisfactory method. The solution used for seepage should ordinarily not be stronger than 1:10. It is, of course, useless to attempt to give soda by rectum when there is diarrhea.

When bicarbonate of soda is given subcutaneously, a 2% solution should be used. When given intravenously, a 4% solution is strong enough. The amount to be given must depend, of course, on the age and size of the patient.

The acetone bodies being formed chiefly from fat in the absence of carbohydrates or when there is a disturbance of the carbohydrate metabolism, another indication is to administer an easily absorbable and utilizable carbohydrate. Such a carbohydrate is glucose or dextrose. This may be given by mouth or by rectum. It is usually advisable to give the soda, both by mouth and by rectum, in a 10% solution of dextrose. In urgent cases dextrose may be given intravenously in the strength of 2½% of dextrose in normal saline solution. Kahlbaum's is the only readily available pure dextrose. When the vomiting stops and the patient begins to improve, the carbohydrates may be added in the form of the cereal waters or jellies, to which one of the various maltose-dextrin mixtures or lactose may be added.

Practically, the usual treatment is the administration of a 5% or 10% solution of bicarbonate of soda in a 10% solution of dextrose freely, both by mouth and rectum. In my experience the immediate and thorough cleaning out of the intestinal tract has seemed to have more effect on the outcome in those cases of acid intoxication secondary to infections or to diseases of the intestinal tract than any other single procedure.

It must be remembered that this treatment is simply for the condition of acid intoxication, and that this condition is not a primary, but a secondary one. Therefore, even if the condition of acid intoxication is relieved, the original causative disease remains. The patient may die, therefore, even after the acid intoxication is cured.—Boston Medical and Surgical Journal. April 20, 1916.

### THE USE OF PITUITRIN IN LABOR.

By Collin Foulkrod, M.D., Philadelphia.

Obstetrician to the Presbyterian Hospital; Demonstrator of Clinical Obstetrics in Jefferson Medical College.

You will observe from the title that we are limited to the action of the drug during labor. Time does not allow for more.

I am still conservative.

It has been of especial interest to read the reports published during the past year by men who have used the drug extensively, and while their results differ from each other, and from my experience, a careful scientific conclusion cannot be reached without considering everything which is accurate in these reports.

The extent to which it has been used can best be judged by a reference to the following papers:

J. L. Bubis, M.D., in Surgery, Gynecology and Obstetrics for November, 1915, reaches the following conclusions: "Pituitrin is an ideal oxytoxic, in that it is safe when properly used. It may be given in any stage of normal labor. Small doses influence the pains and can do very little harm, but with large doses the uterine contractions are beyond control. The infant mortality and the morbidity of the mothers are much greater when large doses are given."

In this paper he reports three cases of disaster early in the use of the drug: two children were lost and one mother torn badly.

An enthusiastic paper read by Samuel W. Bandler before the last meeting of the American Association of Obstetricians and Gynecologists, and published in the January issue of the American Journal of Obstetrics and Diseases of Children, gives these points:

" 'When the head is well molded and fixed in and through the brim of the pelvis.' This phrase evidences my conviction as to one of the essential preliminaries which ought to obtain in primigravidae before pituitary extratc may be used by the general medical man. Whatever the stage this rule does not hold good in women who have had one or more children."

The paper closes with these words: "Practically all men are against its use in the first stage. I assert that in this stage, under proper conditions, it is of the greatest aid in furthering the progress of labor."

Four men discussed this paper; two were opposed to the drug, one having seen a rupture of the uterus from its use, and two agreed with Dr. Bandler.

Ingraham and Chase in Colorado Medicine, vol. xii, 1915, page 190, report their observations on the use of pituitary extract in 44 obstetrical cases.

Pituitary extract is variable in strength.

The individual patient reacts differently to the drug, and therefore caution should be your rule until thorough acquaintance with the possible dangers in each patient assures you of a safe procedure.

Pituitary extract should not be given by the nurse in the absence of the physician; it should not be given and the physician leave the patient.

The only two points upon which all observers agree are, first, that the ampoules on the market are entirely too large to be put into the hands of the general practitioner for house use, and as a standard dosage a full ampoule should never be used as one dose.

At the present we are using onethird of an ampoule, usually five minims.

Most men agree that by itself pituitrin is ineffectual in producing labor when no pains are present.

A consideration of the use of pituitrin must include the causes for which the drug is advanced.

1. Uterine inertia.
2. To control hemorrhage.
3. To differentiate true from false labor pains.
4. To induce labor.
5. To shorten the time of suffering of the woman in normal labor.

6. To enable the doctor to get to the opera, or to the medical meeting.

(6) Let us take up the last first. Is it justifiable to shorten labor, or to induce labor, that the practice of obstetrics may in any manner be regulated? For example, the medical man may have another patient needing him badly. The answer to such a question rests with each individual case, and in some instances it may be necessary to give some of the facts to the husband before doing so. If a safe drug is found I am sure that both patient and physician would be glad to use it.

(5) Is it justifiable to use pituitrin to shorten the time of suffering of a patient who is proceeding normally? From the standpoint of suffering, I am in hopes that the next paper on nitrous-oxide anesthesia will answer the query.

(4) It is the experience of most men that pituitrin by itself fails to induce labor, although Bandler advances the theory that the secretion of the pituitary gland, liberated at a certain time or working in harmony with some other glandular secretion, is the cause of labor.

One patient of my own will serve as a typical illustration:

A multipara, delivered in her first labor by the family physician of a living child, in which labor he expressed the idea that he did everything himself.

Sent to me for second labor. I found it necessary to use bag and manual dilatation to dilate a rigid cervix, and with absolute uterine inertia was compelled to do a hard forceps. This child lived about two weeks and died in convulsions.

The third pregnancy the women went to a maternity hospital at term, not in labor; was given on two occasions each day for two days one-half ampoule of pituitrin, and no other drug or mechanical attempt to produce labor.

Her pains, not severe, lasted after each dose a varying time from two to three hours, then completely subsided. She was allowed to rest for one day to see if labor would supervene. On the next day f℥ij of castor oil was given, and she expelled the child almost before I could reach the hospital.

If used after labor has been started with bougies or gauze then it seems to produce an effect. Such use, however, falls under the guidance of the general rules we shall deduce later.

(3) To differentiate true from false labor pains, so strongly do some men believe that pituitrin is inert when true labor is not present that they use a small dose of the drug to increase the pain, which it accomplishes in true labor, but not in false.

(2) To control hemorrhage after labor.

It has been my practice to give an oxytocic drug as soon as the placenta is out of the cervix or mayhap in the lower segment.

Because of the inability of some patients to absorb ergot from the stomach, in hospital practice I have given it hypodermically. Recently I have been impressed with the fact that even under the skin I could not say definitely there was any marked action; therefore, at least one-half of the cases have received either one-half an ampoule, or, in some instances, as much as an ampoule of pituitrin. The results have been variable. The one thing which stands out most prominently in this series of cases is that the only patient in whom I have seen in ten years a true postpartum hemorrhage was one in which pituitrin was used instead of ergot.

My conclusions are that for such a purpose pituitrin gives a most rapid series of contractions, which may soon exhaust the uterus, and that you may or may not have severe hemorrhage afterward unless the pituitrin is reenforced by the slower long-acting drug, ergot.

Edgar in his paper on the drug in 1913, before the American Gynecological Society, reports giving it in Caesarian section, as contrasted with ergot, and he says: "Then again the pituitary extract patient passed a large clot from the uterus twelve hours after operation, indicating at least that the action of the pituitary extract was not permanent."

(1) Uterine inertia. Just what is uterine inertia? In a hospital clinic where Caesarian section was applied as the cure for those cases in which the force of the uterine contractions was not sufficient to engage the head, I was allowed the privilege of examining the uteri of such cases as needed hysterectomy. I was astonished at the unsuspected cases of fibroid uteri and of another condition which we came to call fibrosis of the uterus, in which concealed and incipient fibroids were the cause of the inability to secure good muscular force.

If this in an unknown percentage of cases is the cause of uterine inertia, are we justified in using pituitrin as an oxytocic? I am sure my judgment is against such a measure whether the head is in the pelvis or not.

Uterine inertia due to toxemia of the mother or death of fetus in utero. Here, again, we are unable to control the severity of the contraction after it has left out hands, and I have seen such a uterus rupture upon the slightest provocation.

In uterine inertia due to a tired muscle after a sturdy long first stage, when the contractions have been good, if a complete rest is secured before giving and the head out of the cervix in the pelvis, if ether and forceps are decried by the family, pituitrin may be used cautiously.

Where the uterus gives evidence of beginning to tire out and the exhaustion depicted above has not been reached, and the same situation exists as to the presenting part and the cervix, then small doses of pituitrin may be tried.

My position and conclusions are then practically the same as deduced by Edgar in 1913 and presented in his paper to the American Gynecological Society that year.

Pituitrin must not be used unless the presenting part is definitely engaged in the pelvis and the largest diameter has passed through the plane of the inlet, and then only if the outlet has been carefully measured for any contraction of the transverse diameter. This relates to multiparae as well as primiparae. It has been our experience that after several normal labors a woman may develop a child too large to pass through a normal pelvis.

I consider pituitrin in the same class as ergot, and as at present going through the same class of experiments as ergot did before it came to have a definite settled use.

In common with other men I have seen one-half an ampoule give such a tetanic contraction of the uterus that I feared rupture, or if not that, then death of the fetus from contraction of the placental site.

I have seen severe shock from one ampoule of pituitrin: in one instance of a multipara, with the head in the pelvis out of the cervix, the shock was so pronounced that labor was set aside entirely until I relieved her with forceps at the end of two hours, fearing for the child.

The dangers to the child must not be minimized; they seem to come from the secondary action through tetanic contractions more vigorous or more prolonged than nature intended, cutting off normal circulation of the fetus through some interference at the placental site.

It is yet to be proven that pituitrin is any safer than ergot used in the same proportionate scale of dosage.—The Therapeutic Gazette, May 15, 1916.

4005 Chestnut St.

References.

J. L. Bubis, M.D.: Surgery, Gynecology and Obstetrics, November, 1915, p. 659.

S. W. Bandler M.D.: Am. Jour. Obs. and Dis. of Women and Children, January 1916, p. 97.

C. B. Ingraham, M.D.: Surgery, Gyncology and Obstetrics, November, 1915, p. 546.

P. M. Chase, M.D.: Surgery, Gynecology and Obstetrics, November, 1915, p. 546.

J. C. Edgar, M.D.: Trans. Am. Gyn. Society, 1913, p.

J. J. Mundell, M.D.: Am. Jour. Obstetrics and Dis. of Women and Children, February, 1916, p. 306.

### AN UNUSUAL AUSCULATION METHOD.

By Felix W. Garcia, M.D., St. Louis, Mo.

About a year ago, while the writer was ausculating a tubercular chest, a member of the family was walking about on the adjoining kitchen floor, and the writer noticed that the transmission of the sound of the footsteps was clear and distinct through the consolidated lung area. It was found easier to mark out the area of consolidation through this method than through the usual procedure. The transmission of the sound of footsteps was remarkably clear through the listening stethoscope. The house was on a fuiet street, on the second floor. The flooring ran the length of the house, and not crosswise. There was a small thing rug on the floor, not covering the entire area, but leaving a bare area next to the wall. The patient was in a wooden bed, and the walker wore ordinary street shose. The writer mentions these details because during the year several total failures to secure such results occurred because of diqering house construction, caused poor acoustic conditions, and interfered in the transmission of sound through the consolidated lung.

In another case the use of this method enabled me to differentiate absolutely between a lobar pneumonia and pleuritic effusion, and aspiration showed an exudate of five ounces—quite an ifty diagnosis. The differentiation showed on change of position, the sharp transmission of sounds becoming peculiarly blurred when the fluid effusion interposed between the pneumonic lung and the chest wall.

Under our present system of diagnostic methods we use sounds generatde within the chest itself, corroborated by percussion. Percussion is not direct, it is redirect. Its production depends upon the artistic ability peculiar to the individual diagnostician and dependent upon wrist expression, and, somewhat, upon the line of musical ability. The listening ear, is some distance from the chest, and the percussion sound, travels through the air, subject to interference of outside and mixing sounds. Moreover the unaided ear must be relied upon to catch and record the particular identity of the sound tones thus received. Morever, the slightest change of the position of the finger on the chest or movement of the patient will alter the percussion note. Few men have the ability to sharply demark the margin of a consolidated area; and, when these have ability to localize by percussion very small spots of consolidation and to arrive at early and precise recognition of minute spots of consolidation, then these spots are so large that future favorableness of prognosis is conjectural. By utilizing the patient's body as a medium for the transmission of osund-waves generated without the body, it follows, according to the fundamental law of acoustics, that the sound waves thus transmitted will be delivered in intensity according to the density of the lung tissue. Consolidation thus becomes the point of delivery, or point of intensity of sound-waves, in exact proportion to its "hepatization." The stethoscope applied to the consolidated spot, transmits the sound waves to the ear directly, free from adventitious sounds. In one case of small apical consolidation, the writer was able to recognize the dense area, without any trouble, by this method.

Of course, the experimentation of this method has been very crude, owing to the limitation of the material, and to the fact that the patient's home is not the best place for scientific experimentation, and that also walking is a crude method of starting sound-waves. Many failures have been met, but, under certain conditions the delivery of sound-waves generated by the impact of footsteps, without the body; have been delivered through the stethoscope to the writer's ear clearly and truly.

The trueness of sound waves is, of course, a vital factor. Mixing sounds are bound to confuse the diagnosis, and the writer has studied the truneness of sound waves thus delivered, and made it the high point of consideration. In my opinion, the trueness of tone by this method is far superior to the trueness of note by percussion, resting as it does upon fewer factors and more immovable fatcors. The patient's body, the sound producing factors, and the diagnostician listening with the stethoscope record fewer changes of position or movement than are found by the percussion method, and, moreover, ts directness is simpler than the indirectness or rather redirectness of percussion.

The writer believes that sound-waves, transmitted through the body, require no education upon the part of the diagnostician in order to be recognized. The sound-waves is delivered true and distinct with a slight feeling of impact.

As regards cavity sounds, I have been unable to study this condition as modifying sound transmission, but have devoted my study to the effect wave upon consolidation. That amphoric breathing, aegiphony, and other pathologic chest sounds, are subject to the laws of acoustics, and that their existence within the body will so modify sound-waves transmitted through the body as to make diagnosis more sure and facile, must be determined by future and better environed investigation.

Wooden beds were, in the writer's studies, the best medium of transmission. On brass or iron beds results were poor. Curiously the patient seated in bed, enveloped in bed clothing, delivered the sound-waves through the consolidation, as well as he did in a wooden chair. Sound waves were easily recognized through a thin undershirt. In fact, almost no exposure of the patient is required.

In studying the theory of this method, it is incontestably true, that with proper sound producing factors, the pitch of sound-waves can be absolutely governed and recorded. Also that the speed and tone of sound-waves can be rushed through the body, in volume of immense variation, without conceivably the slightest injury to the patient, thus making minute spots of consolidation, perfectly plain, though imperceptible to our customary and feeble method of percussion, in fact, giving rein to fancy, consolidated spots may be canned and recorded. However, as speculation enters, the writer will cease, hoping the profession will develop the few meager facts herein presented.—The Medical Fortnightly and Laboratory News.,

3109 S. Grand Avenue.

